# Response Surface Modeling and Parameter Optimization of Microgroove Depth in Water-Jet-Guided Laser Machining of L605 Alloy

**DOI:** 10.3390/mi17050550

**Published:** 2026-04-29

**Authors:** Shimin Yang, Yugang Zhao, Qilong Fan, Li Guo, Zhi Qi, Kai Xing, Yusheng Zhang

**Affiliations:** School of Mechanical Engineering, Shandong University of Technology, No. 266 Xincun West Road, Zibo 255049, China; ysmsdut@126.com (S.Y.); fql0317@126.com (Q.F.); guoli3225@126.com (L.G.); sdtuqizhi@126.com (Z.Q.); sdlgxingkai@126.com (K.X.); zys19862734240@126.com (Y.Z.)

**Keywords:** water-jet-guided laser, L605 alloy, microgroove depth, response surface methodology

## Abstract

L605 cobalt-based superalloy is a typical difficult-to-machine material because of its high strength, pronounced work hardening, and low thermal conductivity. To improve the microgroove machining performance of this alloy, a self-developed water-jet-guided laser (WJGL) system equipped with a multi-focus lens was employed, and single-factor experiments together with a Box–Behnken response surface design were conducted to investigate the effects of laser power, pulse frequency, water pressure, and feed speed on microgroove depth. The results showed that microgroove depth increased with laser power, decreased with pulse frequency and feed speed, and first increased and then decreased with water pressure. Analysis of variance demonstrated that the developed quadratic regression model was significant and fit the data well. A recommended parameter combination of 274.9 W laser power, 3334.9 Hz pulse frequency, 1.636 MPa water pressure, and 0.107 mm/s feed speed corresponded to a predicted microgroove depth of 621.2 μm. Validation experiments yielded an average microgroove depth of 600.2 μm, with a relative error of 3.4%, indicating that the model can be used for microgroove depth prediction and parameter selection in WJGL machining of L605 alloy and may provide guidance for future multi-objective optimization considering both machining quality and efficiency.

## 1. Introduction

L605 cobalt-based superalloy (Haynes 25) exhibits excellent high-temperature strength, oxidation resistance, corrosion resistance, and wear resistance and is therefore widely used in aero-engine hot-section components, chemical equipment, and biomedical devices, particularly implantable devices such as cardiovascular stents [1,2]. However, L605 and related superalloys are regarded as typical difficult-to-machine materials because of their high strength, pronounced work-hardening tendency, and relatively low thermal conductivity. As a result, conventional mechanical machining of these materials often leads to rapid tool wear and poor surface integrity.

Laser machining is a non-contact manufacturing process that avoids the tool wear and workpiece deformation associated with conventional mechanical machining and has become an important approach for processing difficult-to-machine materials such as superalloys. Sudheer et al. [3] investigated the microfabrication of L605 cobalt–chromium cardiovascular stent tubes using a modulated pulsed Nd:YAG laser. Using acousto-optic modulation, they achieved precise control of laser power, thereby enabling high-precision cutting without damaging the material. Zhu et al. [4] studied the structural characteristics and formation mechanisms of pure molybdenum after laser machining and laser water-assisted machining. Their results showed that water-assisted laser machining increased the cooling rate, suppressed recast-layer formation, and reduced material oxidation. Zhang et al. [5] proposed a combined process of laser machining and high-temperature chemical etching for fabricating film holes in IN718 nickel-based superalloy, thereby mitigating the recast layer and heat-affected zone commonly observed in conventional laser machining while improving both hole quality and machining efficiency. In addition, water-jet-guided laser machining has shown advantages in reducing heat-affected zones and improving surface integrity in difficult-to-machine materials, as demonstrated in studies on CFRP machining [6,7].

In recent years, extensive research has been conducted on the application of water-jet-guided laser machining. Subasi et al. [8] investigated the application of WJGL to the microdrilling of Inconel 625 alloy. More recently, systematic studies have been reported on WJGL micro-hole drilling of aerospace alloys and Cf/SiC composites, further confirming the applicability of this process to different difficult-to-machine materials and micro-feature geometries [9,10]. The experimental results showed that drilling efficiency decreased significantly with decreasing hole diameter and increasing drilling depth. In addition, plasma shielding, the cooling effect of the water jet, and splash-back were all found to influence material removal efficiency. Zhao et al. [11] employed WJGL to fabricate microgroove structures in Inconel 718 nickel-based alloy and analyzed the effects of water-jet velocity and laser energy density on groove depth, groove width, and burr size. Their results indicated that laser energy density exerted a greater influence on groove geometry than water-jet velocity. Yu et al. [12] investigated the drilling characteristics of GH4169 superalloy using WJGL and analyzed the effects of laser power and scanning speed on hole-wall morphology, surface roughness, and recast-layer formation. Teng et al. [13] studied the thermal damage mechanism in WJGL machining of K424 superalloy and demonstrated the significant advantage of this process in suppressing thermal effects. In addition to process applications, recent studies have focused on the optical coupling and beam-focusing characteristics of WJGL systems, including coupling tolerance, focusing behavior, and multi-focus strategies for improving machining efficiency in superalloy cutting [14,15,16]. Although WJGL studies have been reported for Inconel 718, GH4169, K424, and CFRP, the machining behavior and parameter–response relationship for L605 cobalt-based superalloy microgrooves have not been systematically clarified. Therefore, the present study investigates the effects of process parameters in water-jet-guided laser machining of L605 alloy.

To address the machining difficulties associated with cobalt-based superalloys, a multi-focus lens was employed in this study to replace the conventional single-focus lens, thereby dispersing the energy density in the focal region and enabling stable water–laser coupling under high-power input conditions. The use of this lens broadened the processing window, thereby enabling the application of higher laser power and improving the machining capability. Based on this design, a self-developed water-jet-guided laser (WJGL) system was used to perform microgroove cutting experiments on L605 alloy plates. The effects of key process parameters on microgroove depth were systematically investigated, and response surface methodology was then employed to optimize the machining parameters. This study may provide guidance for parameter selection in WJGL machining of L605 alloy. It should be noted that the present study is limited to single-objective optimization of microgroove depth. Microgroove cutting depth can serve as an indicator of machining capability, whereas other quality indicators should be investigated in future studies after a sufficient machining capability has been ensured.

## 2. Principle of Water-Jet-Guided Laser Machining

**Figure 1:**
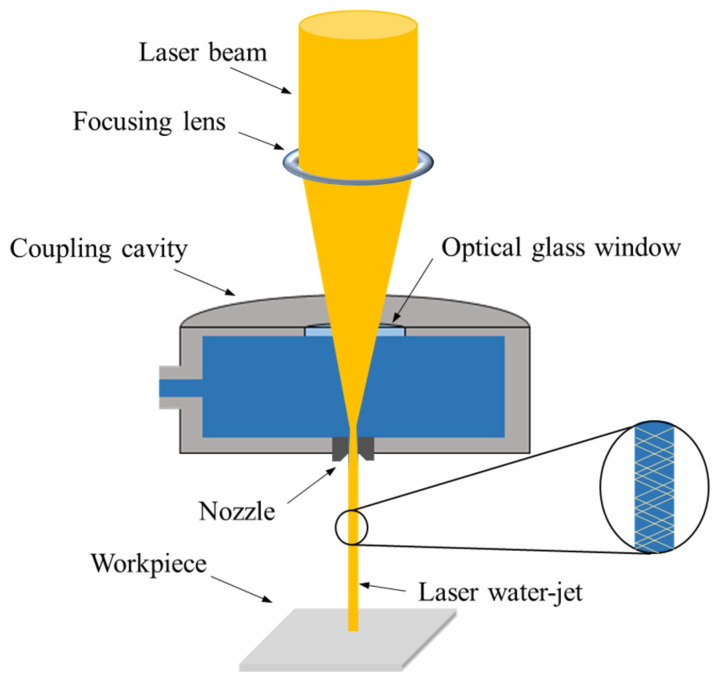
Schematic of the water-jet-guided laser machining principle.

Water-jet-guided laser (WJGL) machining is an advanced hybrid machining technology developed for precision material processing. It employs a fine water jet to guide the laser beam to the workpiece for precision machining. This process combines the advantages of laser machining, including non-contact processing and the absence of mechanical stress, with the cooling and flushing effects of the water jet, thereby enabling machining with reduced thermal damage and improved surface quality [17,18]. The machining principle of WJGL is illustrated in Figure 1.

Optical coupling in WJGL is achieved through total internal reflection. Because water and air have different refractive indices, total internal reflection occurs when the incident angle of the focused laser beam is greater than or equal to the critical angle for total internal reflection at the water–air interface. Under this condition, the laser beam is confined within the water jet and propagates along it in a guided manner through total internal reflection. In this way, the water jet acts as an optical waveguide that transports laser energy to the workpiece surface and enables material removal.

## 3. Experimental Design

### 3.1. Experimental Setup and Materials

**Figure 2:**
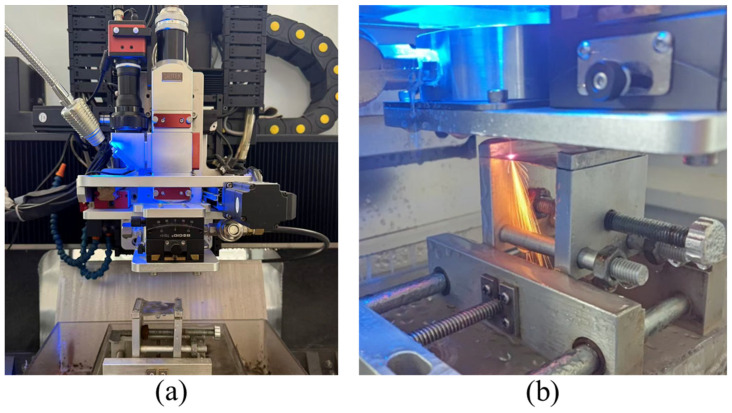
(**a**) Water-jet-guided laser machining system and (**b**) WJGL machining of L605.

**Figure 3:**
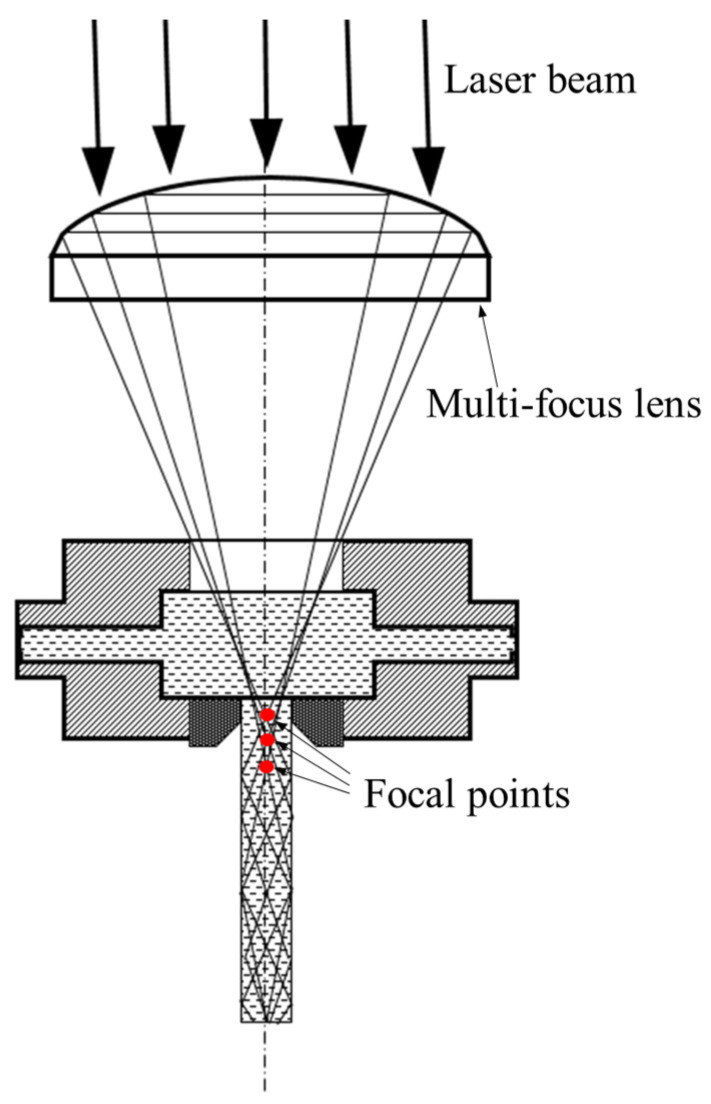
Multi-focus lens coupling schematic diagram.

The microgroove cutting experiments were conducted using a self-developed WJGL system, as shown in Figure 2. The system mainly consisted of a laser–water-jet generation unit, a high-pressure water supply system, a laser source, a control system, and an alignment and monitoring system. The laser source was a YLR-2000-WC (IPG, Marlborough, MA, USA) laser with a wavelength of 1064 nm and a maximum output power of 2000 W. The water supply system pressurized deionized water to generate a stable water jet, with an adjustable pressure range of 0–20 MPa. The three-axis stage had a travel range of 500 mm × 300 mm × 300 mm and a repeat positioning accuracy of ±3 μm. The water–laser coupling device was mounted on the *Z*-axis, whereas the workpiece was fixed on a two-dimensional platform, thereby enabling complex machining trajectories. In this system, a multi-focus lens was employed instead of a conventional single-focus lens. When a parallel laser beam passes through the multi-focus lens, multiple focal points are formed along the optical axis. As a result, the laser energy is distributed among multiple focal points, and the energy density at each focal point is significantly reduced. This design can effectively prevent water-jet breakdown under high laser-power conditions. A schematic diagram of the coupling structure is shown in Figure 3.

**Figure 4:**
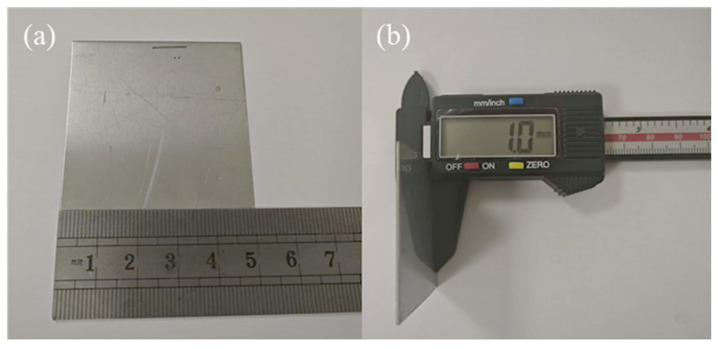
(**a**) Width of the experimental material and (**b**) Thickness of the experimental material.

The workpiece material was an L605 cobalt-based superalloy plate measuring 50 mm × 50 mm × 1 mm. Its chemical composition is listed in Table 1, and its mechanical properties are summarized in Table 2. The experimental material is shown in Figure 4.

### 3.2. Experimental Methods

To investigate the effects of process parameters on microgroove depth in WJGL machining and to determine appropriate factor ranges for the response surface experiments, single-factor experiments were first conducted. Based on preliminary trials, the effects of laser average power, pulse frequency, pulse width, water pressure, feed speed, stand-off distance, and number of scanning passes on machining performance were systematically evaluated. Among these factors, laser average power, pulse frequency, water pressure, and feed speed were identified as the dominant variables affecting microgroove depth and were therefore selected as the main experimental factors. The factor levels and corresponding parameter values are listed in Table 3.

**Figure 5:**
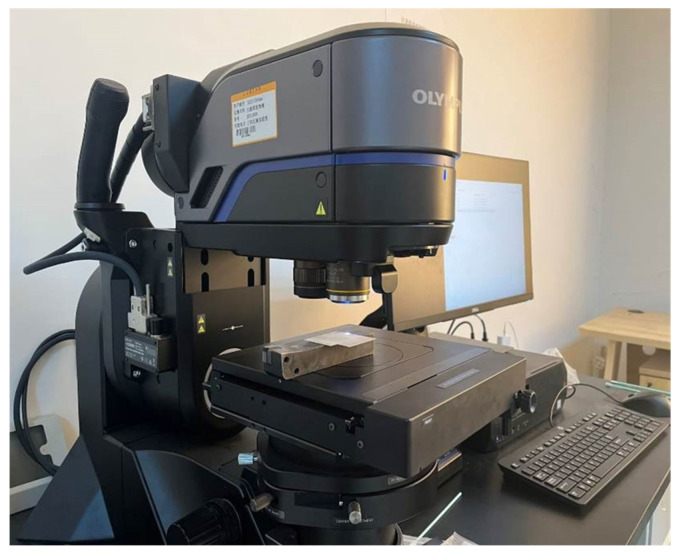
Measurement setup (DSX1000, Olympus).

All other process parameters were kept constant during the experiments: the nozzle diameter was 0.5 mm and the number of scanning passes was 10. Due to nonlinear optical effects, laser energy attenuates during propagation through the water jet, and the attenuation becomes more pronounced as the transmission distance increases. Therefore, to improve laser energy utilization efficiency, the stand-off distance in this study was set to 10 mm. After machining, the sample cross-sections were ground and polished with abrasive paper and then subjected to ultrasonic cleaning. As shown in Figure 5, the cross-sectional morphology of the microgrooves was then examined using a high-resolution digital microscope (DSX1000, Olympus, Tokyo, Japan). For each sample, the final microgroove depth was determined as the average of three measurements obtained from the prepared cross-section. The results in the single-factor plots are presented as mean values with standard deviation error bars.

After the results of the single-factor experiments were analyzed, appropriate parameter ranges were selected for the response surface experiments. Considering that material removal is insufficient at low laser power, whereas excessively high laser power may reduce nozzle service life, the parameter ranges listed in Table 4 were adopted. With microgroove depth as the response variable, response surface experiments were conducted to optimize the WJGL process parameters for L605 alloy. The Box–Behnken design (BBD) is a widely used experimental design in response surface methodology [19,20]. It can significantly reduce the number of experimental runs through combinations of three factor levels (low, medium, and high) while maintaining high efficiency and good model adaptability. Therefore, BBD was employed in this study to design the experiments and establish a mathematical prediction model for microgroove depth.

## 4. Results and Discussion

### 4.1. Single-Factor Experimental Results and Analysis

**Figure 6:**
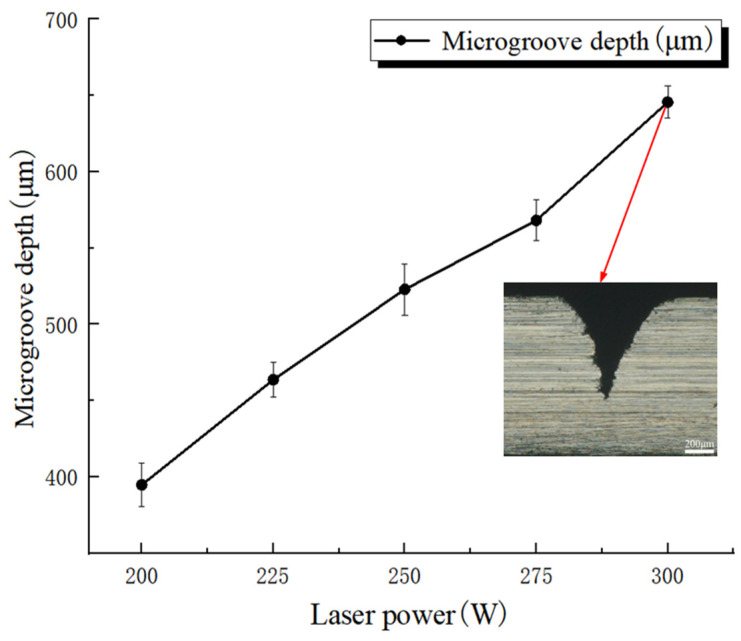
Effect of laser power on microgroove depth.

Figure 6 shows the effect of laser power on microgroove depth. Error bars represent standard deviations based on three repeated measurements. In this set of experiments, the pulse frequency, feed speed, and water-jet pressure were fixed at 5000 Hz, 0.2 mm/s, and 1.5 MPa, respectively, whereas the laser average power was varied from 200 W to 300 W in increments of 25 W. The mean microgroove depths obtained from three measurements were 394.9 ± 14.1 μm, 463.8 ± 11.2 μm, 522.9 ± 16.7 μm, 568.2 ± 13.4 μm, and 645.6 ± 10.5 μm, respectively. The cross-sectional image shows the groove morphology corresponding to the maximum microgroove depth within the investigated range of this factor. The results indicate that, when the other process parameters were kept constant, microgroove depth increased with increasing laser power. This behavior can be attributed to the fact that material removal in WJGL machining mainly depends on laser energy input. The single-pulse laser energy is related to the laser power and can be expressed as Equation (1):

(1) $$\begin{array}{c} E_{p}=\frac{P}{f} \end{array}$$

where $E_{p}$ is single-pulse energy, P is average laser power, and f is laser repetition frequency. When the pulse frequency remains constant, an increase in average laser power leads to a higher energy density, thereby improving the cutting capability. As laser power increases, the energy delivered to the workpiece per unit time rises significantly, making it easier for the material to reach the energy-density threshold required for melting or even vaporization and thereby improving material removal efficiency. In addition, enhanced energy transfer into the interior of the microgroove further contributes to the increase in microgroove depth as laser power increases.

**Figure 7:**
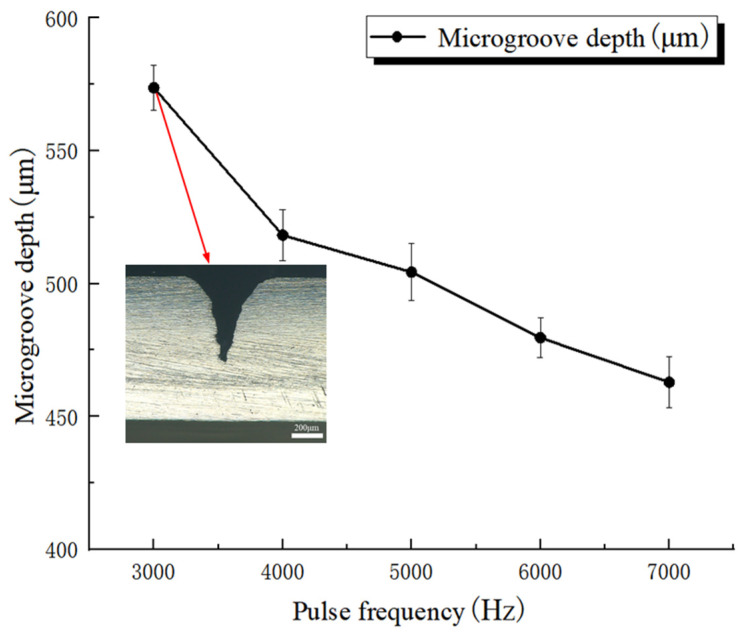
Effect of pulse frequency on microgroove depth.

Figure 7 presents the effect of pulse frequency on microgroove depth. Error bars represent standard deviations based on three repeated measurements. In this group of experiments, the laser power, feed speed, and water-jet pressure were fixed at 250 W, 0.2 mm/s, and 1.5 MPa, respectively, whereas the pulse frequency was varied from 3000 Hz to 7000 Hz. The mean microgroove depths obtained from three measurements were 573.7 ± 8.4 μm, 518.2 ± 9.6 μm, 504.3 ± 10.7 μm, 479.6 ± 7.4 μm, and 462.9 ± 9.5 μm, respectively. The cross-sectional image shows the groove morphology corresponding to the maximum microgroove depth within the investigated range of this factor. The results show that, when the other process parameters remained unchanged, microgroove depth decreased with increasing pulse frequency. This trend can be attributed to the reduction in single-pulse energy at higher pulse frequencies, which weakens the material-removal capability of each pulse and makes it more difficult to exceed the melting threshold of the material. In addition, under high-frequency conditions, the pulse interval becomes shorter, thereby reducing the effective time available for the water jet to remove molten material. As a result, molten debris tends to accumulate inside the microgroove, which further suppresses laser energy transfer in the depth direction. Consequently, microgroove depth decreases as pulse frequency increases.

**Figure 8:**
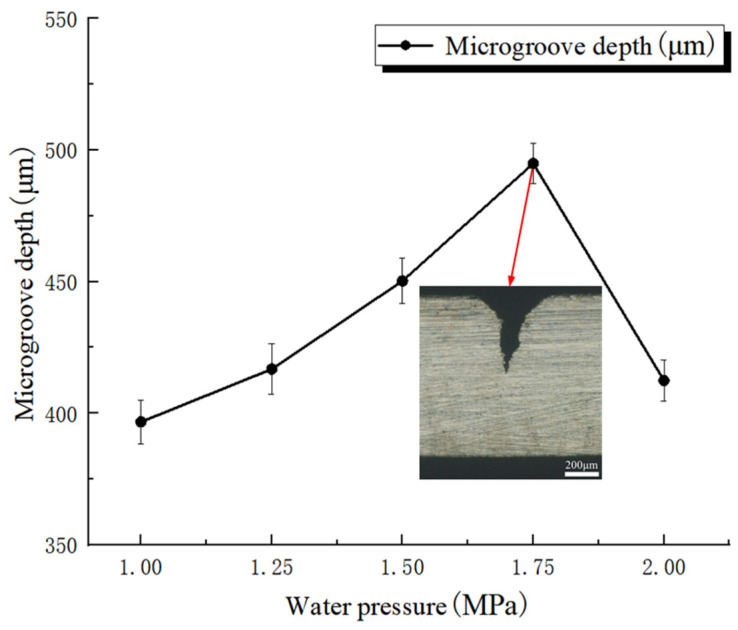
Effect of water pressure on microgroove depth.

Figure 8 shows the effect of water pressure on microgroove depth. Error bars represent standard deviations based on three repeated measurements. In this case, the laser power, feed speed, and pulse frequency were fixed at 250 W, 0.2 mm/s, and 5000 Hz, respectively, whereas the water pressure was varied from 1.0 MPa to 2.0 MPa. The mean microgroove depths obtained from three measurements were 396.7 ± 8.3 μm, 416.8 ± 9.6 μm, 450.3 ± 8.6 μm, 494.9 ± 7.5 μm, and 412.4 ± 7.8 μm, respectively. A representative cross-sectional image corresponding to the maximum microgroove depth within the investigated range of this factor is shown in the inset. The results show that, with all other parameters held constant, microgroove depth first increased and then decreased as water pressure increased. During WJGL machining, water pressure plays an important role by influencing the velocity and flow state of the water jet. As water pressure increases, the water-jet velocity rises and its kinetic energy increases, thereby facilitating the removal of molten material and debris from the microgroove and promoting laser energy transfer toward deeper regions. However, when water pressure becomes excessively high, the water jet may transition from a stable laminar state to a turbulent state, resulting in instability of the water column and a reduction in its laser-guiding capability. Moreover, excessive cooling may remove additional heat from the machining zone, thereby weakening thermal accumulation and ultimately reducing microgroove depth.

**Figure 9:**
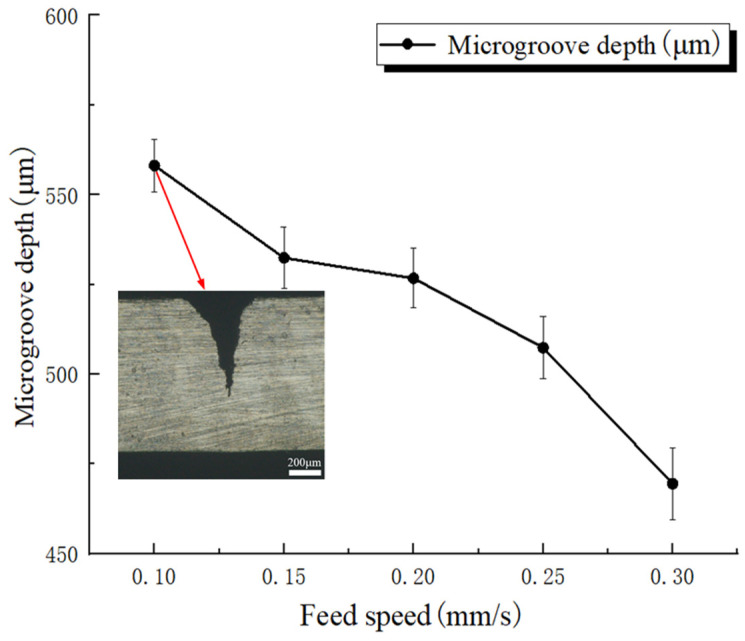
Effect of feed speed on microgroove depth.

Figure 9 illustrates the effect of feed speed on microgroove depth. Error bars represent standard deviations based on three repeated measurements. In this set of experiments, the laser power, pulse frequency, and water-jet pressure were fixed at 250 W, 5000 Hz, and 1.5 MPa, respectively, whereas the feed speed was varied from 0.1 mm/s to 0.3 mm/s. The mean microgroove depths obtained from three measurements were 558.1 ± 7.3 μm, 532.4 ± 8.6 μm, 526.7 ± 8.3 μm, 507.4 ± 8.6 μm, and 469.5 ± 10.0 μm, respectively. The cross-sectional image shows the groove morphology corresponding to the maximum microgroove depth within the investigated range of this factor. The experimental results indicate that, when the other process parameters were kept constant, microgroove depth decreased with increasing feed speed. This behavior can be attributed to the fact that a higher feed speed reduces the energy input per unit length and shortens the laser interaction time within the machining zone. As a result, the material cannot be fully melted or vaporized, leading to a lower material-removal rate and, consequently, a reduction in microgroove depth.

### 4.2. Results and Analysis of the Response Surface Experiments

Microgroove depth was selected as the response variable for the machined L605 alloy plates, and the corresponding experimental results are listed in Table 5. A quadratic polynomial model was employed to fit the microgroove depth data, and the resulting regression equation describing the effects of the main process parameters is given in Equation (2).

(2) $$\begin{array}{c} \begin{array}{c} H=492.96-62.3A-41.83B+11.15C-37.95D+5.25AB-3.25AC+0.25AD\\ +3BC-2.25BD+7.4A^{2}+1.65B^{2}-25.81C^{2}-1.46D^{2} \end{array} \end{array}$$

Analysis of variance (ANOVA) was employed to evaluate the fitting quality of the model, and the corresponding results for microgroove depth are presented in Table 6. The F-value, defined as the ratio of the variance between groups to the variance within groups, was used to assess the overall significance of the model. As shown in Table 6, the model F-value was 63.73, with a *p*-value smaller than 0.0001, indicating that the quadratic regression model can effectively describe the relationship between microgroove depth and the investigated process parameters. All four main effects were significant. Among them, laser power exerted the greatest influence on microgroove depth, followed by pulse frequency and feed speed, whereas water pressure had a relatively smaller but still significant effect. None of the interaction terms reached statistical significance, suggesting that the interactions among the factors were weak within the investigated range. The lack-of-fit *p*-value was 0.8265, which was greater than 0.05, indicating that the lack of fit was not significant and that the model showed good agreement with the experimental data. The coefficient of determination (R^2^) was 0.9846, and the adjusted coefficient of determination (Adj. R^2^) was 0.9691, indicating that the model explained 96.91% of the variation in the response and possessed good predictive capability. The dominant effect of laser power was also consistent with the single-factor experimental results.

**Figure 10:**
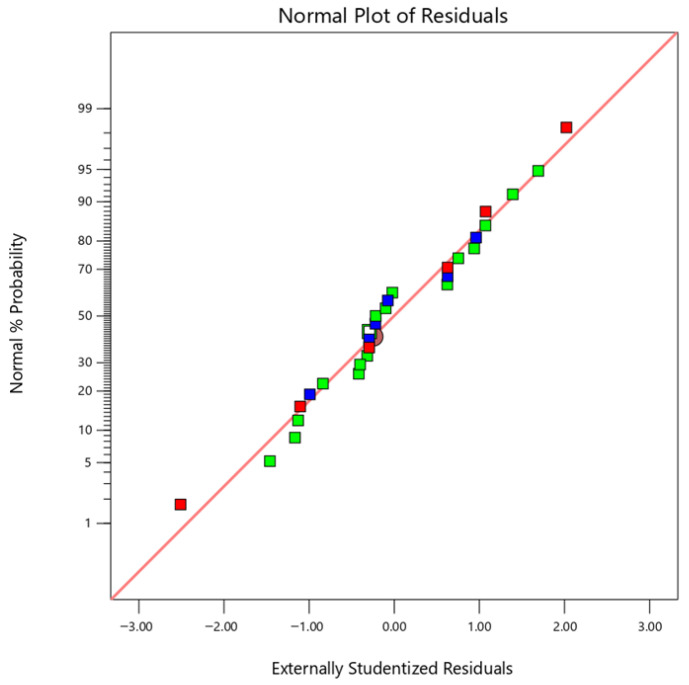
Normal probability plot of the residuals for the predictive model.

As shown in Figure 10, most data points are distributed close to the reference line, indicating that the residuals approximately follow a normal distribution. This result suggests that the normality assumption for the residuals is reasonably satisfied, supporting the validity of the ANOVA results.

**Figure 11:**
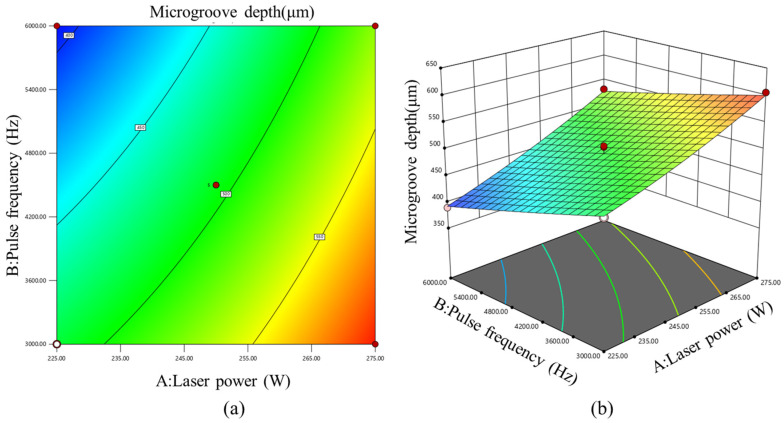
(**a**) Contour plot and (**b**) three-dimensional response surface of the effects of laser power and pulse frequency on microgroove depth.

Figure 11 shows the three-dimensional response surface and contour plot illustrating the combined effects of laser power and pulse frequency on microgroove depth. The surface rises markedly along the laser power axis, indicating that increasing laser power significantly increases microgroove depth. In contrast, the surface declines along the pulse frequency axis, suggesting that increasing pulse frequency is unfavorable for increasing microgroove depth. Moreover, the steeper gradient along the laser power axis indicates that laser power exerts a stronger influence on microgroove depth than pulse frequency, which is consistent with the ANOVA results, where factor A showed higher significance than factor B. Therefore, within the investigated range, higher laser power and lower pulse frequency were more favorable for achieving greater material removal.

**Figure 12:**
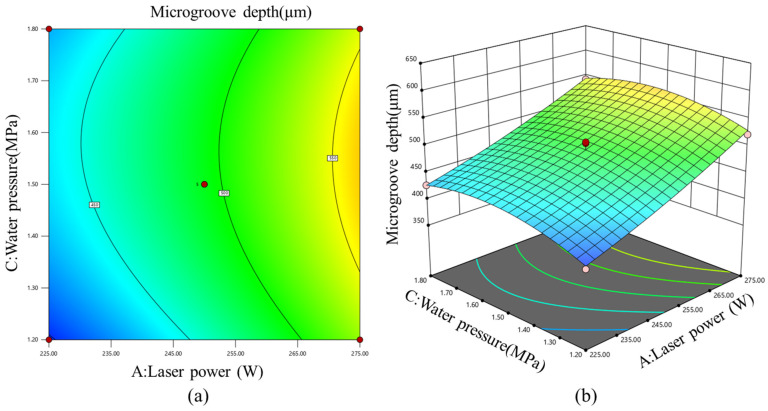
(**a**) Contour plot and (**b**) three-dimensional response surface of the effects of laser power and water pressure on microgroove depth.

Figure 12 presents the response surface and contour plot of laser power and water pressure, illustrating their combined effects on microgroove depth. The three-dimensional response surface shows that microgroove depth generally increased from the low-power/low-pressure region toward the high-power/intermediate-pressure region, indicating a dominant positive effect of laser power and a weak quadratic effect of water pressure. When laser power was fixed at the central level, microgroove depth first increased and then decreased as water pressure increased. This behavior was closely associated with the stability of the water jet. At low water pressure, molten material could not be effectively expelled from the deeper region of the slit, thereby limiting material removal at the bottom of the microgroove. At excessively high water pressure, the stability of the water jet decreased, and the laser-guiding capability of the non-laminar jet was weakened, leading to a reduction in water–laser coupling efficiency.

**Figure 13:**
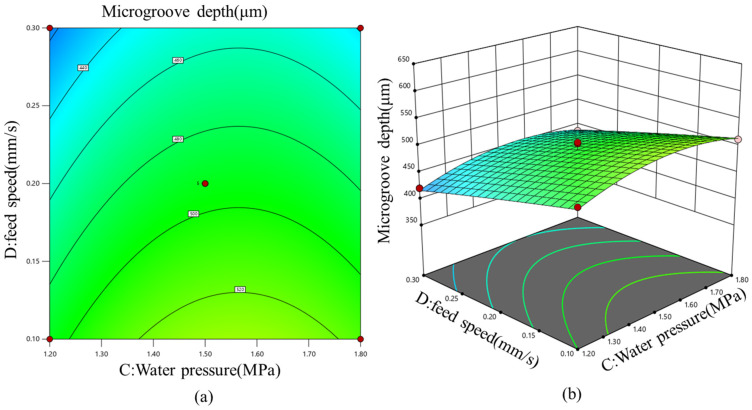
(**a**) Contour plot and (**b**) three-dimensional response surface of the effects of water pressure and feed speed on microgroove depth.

Figure 13 illustrates the response surface and contour plot of water pressure and feed speed, showing their combined effects on microgroove depth. When water pressure was fixed, microgroove depth decreased approximately linearly with increasing feed speed, which was consistent with the single-factor experimental results. This trend indicates that a higher feed speed reduced the laser dwell time in the machining zone, thereby decreasing energy accumulation and lowering the material-removal rate.

### 4.3. Validation of the Optimization Results

**Figure 14:**
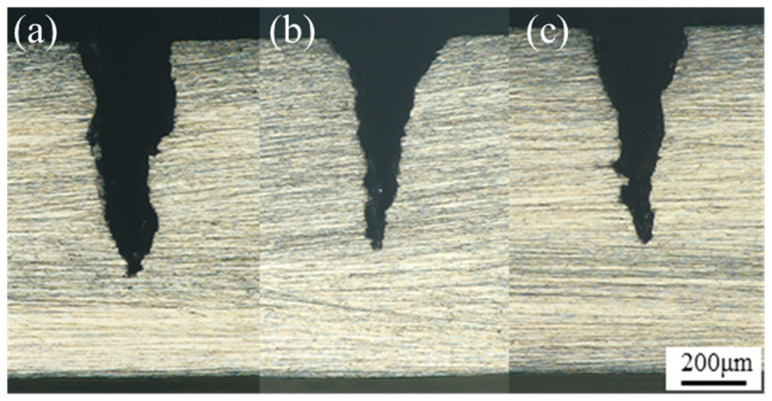
(**a**–**c**) Cross-sectional morphologies of the microgrooves produced under the three validation conditions.

Based on the optimization results of the response surface model, a parameter combination predicted to yield a high microgroove depth was selected for validation. With microgroove depth as the optimization objective, the recommended parameter combination was obtained as follows: a laser power of 274.9 W, a pulse frequency of 3334.9 Hz, a water pressure of 1.636 MPa, and a feed speed of 0.107 mm/s. Under these conditions, the predicted microgroove depth was 621.2 μm. To verify the reliability of the model, three repeated machining experiments were conducted under the selected parameter combination. The cross-sectional morphology is shown in Figure 14. The measured microgroove depths were 610.4 μm, 603.6 μm, and 586.7 μm, respectively, yielding an average value of 600.2 μm. The relative error between the measured average and the predicted value was 3.4%. The experimental parameters and corresponding validation results are presented in Table 7. This deviation was within an acceptable experimental range and may be attributed to measurement uncertainty and slight fluctuations in water-jet stability. These results indicate that the developed model provides good predictive accuracy and reliability for microgroove depth in WJGL machining of L605 alloy.

## 5. Conclusions

(1) Within the investigated experimental range, the significance of the process parameters affecting microgroove depth was ranked as follows: laser power > pulse frequency > feed speed > water pressure. Specifically, microgroove depth increased with increasing laser power, decreased with increasing pulse frequency and feed speed, and exhibited a nonlinear dependence on water pressure, with a relatively favorable pressure range of approximately 1.6–1.8 MPa.
(2) With microgroove depth as the optimization objective, response surface methodology yielded a recommended parameter combination of 274.9 W laser power, 3334.9 Hz pulse frequency, 1.636 MPa water pressure, and 0.107 mm/s feed speed. Under these conditions, the predicted microgroove depth was 621.2 μm. Validation experiments showed that the relative error between the measured and predicted values was 3.4%, indicating that the established model provides good predictive accuracy and reliability.
(3) The results demonstrate that response surface methodology can effectively describe the relationship between microgroove depth and the major process parameters in WJGL machining of L605 alloy and may provide guidance for parameter selection for microgroove-depth control. It should be noted that the present study was limited to the investigation and optimization of microgroove depth, while other important machining-quality indicators, such as groove width, taper, surface roughness, recast layer, burr formation, and heat-affected zone, were not quantitatively evaluated. Further studies are needed to perform multi-objective optimization incorporating both machining capability and machining-quality indicators.

## Figures and Tables

**Table 1:** Chemical composition of L605 material.

Element	Fe	C	Si	Mn	Cr	Ni	W	Mo	P	S	Co
Content (wt%)	1.6	0.1	0.6	1.5	20	10	15	≤1.0	≤0.04	≤0.03	Balance

**Table 2:** Mechanical properties of L605 material.

Tensile Strength (MPa)	Yield Strength (MPa)	Elongation (%)	Brinell Hardness (HB)
996	476	54.7	282–413

**Table 3:** Single-factor experimental factors and levels.

Factors	Level				
	1	2	3	4	5
Laser power (W)	200	225	250	275	300
Pulse frequency (Hz)	3000	4000	5000	6000	7000
Water pressure (MPa)	1	1.25	1.5	1.75	2
Feed speed (mm/s)	0.1	0.15	0.2	0.25	0.3

**Table 4:** Factors and levels used in the Box–Behnken design.

Factors	Level		
	−1	0	1
A—Laser power (W)	225	250	275
B—Pulse frequency (Hz)	3000	4500	6000
C—Water pressure (MPa)	1.2	1.5	1.8
D—Feed speed (mm/s)	0.1	0.2	0.3

**Table 5:** Response surface experimental results.

Run	Factors				Results
	Laser Power (W)	Pulse Frequency (Hz)	Water Pressure (MPa)	Feed Speed (mm/s)	Microgroove Depth (μm)
1	225	4500	1.5	0.3	410.4
2	250	4500	1.5	0.2	489.2
3	250	6000	1.5	0.1	491
4	250	4500	1.8	0.1	512.8
5	250	4500	1.5	0.2	505.2
6	250	3000	1.5	0.3	492.1
7	250	4500	1.2	0.1	496.7
8	250	4500	1.5	0.2	507.4
9	250	6000	1.2	0.2	419
10	250	4500	1.5	0.2	482.8
11	250	6000	1.5	0.3	397.1
12	250	3000	1.5	0.1	577
13	250	4500	1.8	0.3	436.9
14	225	4500	1.2	0.2	392.3
15	275	4500	1.5	0.1	592.4
16	250	3000	1.8	0.2	518.1
17	275	3000	1.5	0.2	605.9
18	275	4500	1.8	0.2	542.6
19	225	6000	1.5	0.2	389.8
20	225	3000	1.5	0.2	484.8
21	225	4500	1.5	0.1	473.3
22	250	6000	1.8	0.2	448.1
23	250	3000	1.2	0.2	501
24	250	4500	1.5	0.2	480.2
25	275	4500	1.2	0.2	521.4
26	250	4500	1.2	0.3	420.8
27	275	6000	1.5	0.2	531.9
28	225	4500	1.8	0.2	426.5
29	275	4500	1.5	0.3	530.5

**Table 6:** ANOVA results for the quadratic regression model of microgroove depth.

Source	Sum of Squares	df	Mean Square	F	Prob > F	Significance
Model	92,041.85	14	6574.42	63.73	<0.0001	Significant
A-Laser power	46,575.48	1	46,575.48	451.51	<0.0001	
B-Pulse frequency	21,000.33	1	21,000.33	203.58	<0.0001	
C-Water pressure	1491.87	1	1491.87	14.46	0.0019	
D-Feed speed	17,282.43	1	17,282.43	167.54	<0.0001	
AB	110.25	1	110.25	1.07	0.3188	
AC	42.25	1	42.25	0.4096	0.5325	
AD	0.25	1	0.25	0.0024	0.9614	
BC	36	1	36	0.349	0.5641	
BD	20.25	1	20.25	0.1963	0.6645	
CD	0	1	0	0	1	
A2	354.72	1	354.72	3.44	0.0849	
B2	17.55	1	17.55	0.1702	0.6862	
C2	4319.34	1	4319.34	41.87	<0.0001	
D2	13.73	1	13.73	0.1331	0.7207	
Residual	1444.18	14	103.16			
Lack of fit	805.67	10	80.57	0.5047	0.8265	Not significant
Pure error	638.51	4	159.63			
Total	93,486.03	28				
R^2^ = 0.9846				Adj. R^2^ = 0.9691		

**Table 7:** Experimental verification results.

Factors	A-Laser Power	B-Pulse Frequency	C-Water Pressure	D-Feed Speed	Predicted Microgroove Depth	Measured Microgroove Depth	Error
Unit	W	Hz	MPa	mm/s	μm	μm	%
Value	274.9	3334.9	1.636	0.107	621.2	600.2 ± 12.2	3.4

## Data Availability

The datasets used or analyzed during the current study are available from the corresponding author upon reasonable request.
